# Identification of anxiety-depressive disorders in paramedics working shift work

**DOI:** 10.1192/j.eurpsy.2023.1013

**Published:** 2023-07-19

**Authors:** D. Brahim, N. Mechergui, I. Youssef, W. Ayed, M. Mersni, S. Ernez, N. Ladhari

**Affiliations:** Occupational pathology and fitness for work, Charles Nicolle Hospital, Tunis, Tunisia

## Abstract

**Introduction:**

Shift work can lead to mental health problems evolving into real anxiety disorders with significant socio-professional repercussions.

**Objectives:**

The purpose of our work was to screen paramedics with shift work for anxiety disorders.

**Methods:**

Cross-sectional study carried out among the paramedical staff of a Tunisian university hospital. The data was collected from a pre-established record sheet. Screening for anxiety-depressive disorders was done using the HAD scale

**Results:**

The study included 158 paramedics. The average age was 36.48 years with female predominance at 70.9%. The average working time was 11.1 8.9 years and the average working time in shift work was 10.27 9.2 years. Of the 13 departments with non-standard hours of work, the main departments represented were resuscitation (n=24; 15.2%), emergencies (n=18; 11.4%) and radiology (n=17; 10.8%). Nurses accounted for 46.2%, manual workers for 23.4% and senior technicians for 19%.Certain anxiety symptomatology was found in 53.2% (n=84) and doubtful symptomatology in 29.1% (n=46) of the general population. Certain depressive symptomatology was noted in 17.1% (n=27) and doubtful symptomatology in 30.4% (n=48) of the general population. Occupational seniority and seniority in shift work were statistically significantly associated with both anxiety (p=0.04/p=0.05) and depression (p=0.05/p=0.006) symptomatology. ). Similarly, this anxiety-depressive symptomatology was associated with the position occupied (p=0.02 / p=0.04) and the assignment department (p=0.008 / p=0.01).

**Conclusions:**

Anxiety-depressive disorders are common among paramedics working shift work in hospitals. Screening consultations in occupational medicine are necessary to detect these disorders early.

**Disclosure of Interest:**

None Declared

